# Incidence and Impact on Quality of Life of Heavy Menstrual Bleeding in Women on Oral Anticoagulant Therapy

**DOI:** 10.1177/10760296241281366

**Published:** 2024-08-30

**Authors:** Naseerah Hassan, Elise Schapkaitz, Haroun Rhemtula, Nolukholo Ncete

**Affiliations:** 1 Dept. of Molecular Medicine and Hematology, Charlotte Maxeke Johannesburg Academic Hospital, National Health Laboratory System Complex and University of the Witwatersrand, Johannesburg, South Africa; 2 Dept. of Obstetrics, Charlotte Maxeke Johannesburg Academic Hospital and University of the Witwatersrand, Johannesburg, South Africa

**Keywords:** heavy menstrual bleeding, warfarin, rivaroxaban, iron deficiency, pictorial blood loss assessment chart, menstrual bleeding questionnaire

## Abstract

**Introduction:**

Heavy menstrual bleeding affects up to two thirds of women on oral anticoagulation. The rates of heavy menstrual bleeding, its impact on quality of life and associated risk factors in women attending anticoagulation clinics in South Africa are largely unknown.

**Materials and Methods:**

A prospective cohort study was performed over an eight-month period in women on Warfarin (n = 30) and Rivaroxaban (n = 27) for a median [interquartile range] duration of 15.5 [78.0] months attending an anticoagulation clinic in Johannesburg, South Africa. Heavy menstrual bleeding was assessed over one menstrual cycle using the validated pictorial blood loss assessment charts (PBAC) and the menstrual bleeding questionnaire (MBQ).

**Results:**

In this population of predominantly African ethnicity, with a median age of 39 [8] years, 39 (68.4%) women experienced heavy menstrual bleeding, defined as a PBAC score of >100. Median cycle length on anticoagulation and MBQ scores were significantly higher among women with a PBAC score of >100 (p > 0.05). Univariate analysis identified Rivaroxaban as a risk factor for heavy menstrual bleeding (OR 5.03, 95% CI 1.40–18.12). Heavy menstrual bleeding required treatment in 29 (74.4%) women which included management of iron deficiency, anti-fibrinolytics, modification of anticoagulation and hormonal contraception.

**Conclusion:**

Heavy menstrual bleeding was associated with a considerable negative impact on quality of life. This was most significant for women on Rivaroxaban as compared to Warfarin. It is essential to monitor and appropriately treat heavy menstrual bleeding in at risk women on anticoagulant treatment.

## Introduction

Heavy menstrual bleeding affects approximately 15–30% of women in the reproductive age group.^1^ Objectively, heavy menstrual bleeding is defined as the quantitative measurement of more than 80 ml of blood loss per cycle. The International Federation of Gynaecology and Obstetrics (FIGO) defines heavy menstrual bleeding as an increase in frequency, irregularity, duration and volume of menstrual blood loss as well as intermenstrual bleeding.^2^ This is based on visualization of clots of more than one inch in diameter, a low ferritin of <20 ng/mL and/or the need to change sanitary pads at least every hour.^3^ In clinical practice, accurate quantification is difficult. A more practical definition which has been suggested is heavy menstrual bleeding that affects a woman's physical, emotional and social quality of life.^4^

Oral anticoagulant treatment for the management and prevention venous thrombo-embolism (VTE) is associated with an increased risk of bleeding, including heavy menstrual bleeding.^5^ While, heavy menstrual bleeding has been reported to affect up to two thirds of women on anticoagulation, few studies, however, have assessed heavy menstrual bleeding specifically as an outcome of anticoagulant use.^6^ A retrospective study conducted in 104 women in Belgium, reported heavy menstrual bleeding in 70.2% of anticoagulated women.^7^ A recently conducted case-control study in the United Kingdom reported a significantly increased median duration of menstrual bleeding from five to six days in anticoagulated women (p < 0.05). This was associated with a negative impact on quality of life, including avoiding social activities, bleeding that soaked outer clothing and unpredictable cycles, as compared to the control group of women.^8^

Oral anticoagulants include vitamin K antagonists and direct oral anticoagulants (DOAC). DOAC have a lower risk of major bleeding, in particular intracranial haemorrhage, as compared to vitamin K antagonists. According to pooled safety data from phase 3 randomized trials in patients with VTE, DOAC were associated with a lower risk of major bleeding (odds ratio [OR] 0.6, 95% confidence interval [CI] 0.5-0.9), and a similar risk of gastrointestinal bleeding (OR 1.1, 95% CI 0.6-2.0) as compared to vitamin K antagonists. In patients with non-valvular atrial fibrillation the risk for gastrointestinal bleeding, however, was higher with DOAC.^9^ Following the wide-spread use of the DOACs, case series and small cohort studies emerged also reporting increased rates of heavy menstrual bleeding compared with vitamin K antagonists.^7,8,10^ In particular, an increased risk of heavy menstrual bleeding was described in the subgroup of women treated with factor Xa inhibitors as compared to women treated with vitamin K antagonists.^8,11^ Subsequently, research has been performed to determine the risks associated with different classes of factor Xa inhibitors. An increase in heavy menstrual bleeding in women on Rivaroxaban, as compared to Warfarin has been described.^12^ This finding was confirmed in post hoc analyses of the EINSTEIN trial which reported increased rates of heavy menstrual bleeding for Rivaroxaban (hazard ratio, 2.1; 95% CI, 1.6–2.9) as compared to vitamin K antagonists.^13^ An American study, RAMBLE (NCT02829957) is currently investigating the rates of heavy menstrual bleeding between Rivaroxaban and Apixaban to determine whether there is indeed a class effect.

Heavy menstrual bleeding is underreported by women attending anticoagulation clinics.^14^ Women are often unaware of the extent of menstrual blood loss and often seek medical care only when their quality of life is affected or as a result of iron deficiency anaemia.^15^ According to a Spanish study, patients’ primary concerns were the effects on functional, professional, social and family responsibilities.^16^ Iron deficiency anaemia is an important complication of heavy menstrual bleeding which can lead to reduced cognitive ability, physical strength and immunity. In particular, iron deficiency anaemia impairs attention, memory, and learning. Treatment options including hormone contraception, anti-fibrinolytic agents, haematinics or uterine surgical interventions which can result in an improvement of symptoms.^17^ However, data comparing treatment options, to guide management decisions in clinical practice, are lacking.

The rates of heavy menstrual bleeding, its impact on quality of life and associated risk factors in women attending anticoagulation clinics in South Africa are largely unknown.^18^ A prospective cohort study was performed to assess the incidence and symptoms of heavy menstrual bleeding using the validated pictorial blood loss assessment charts (PBAC) and the menstrual bleeding questionnaire (MBQ). Moreover, this study aimed to identify risk factors for heavy menstrual bleeding on oral anticoagulation in order to improve the management of this subgroup.

## Methods

### Study Design and Population

A prospective study was performed over an eight-month period from 1 November 2023 to 30 June 2024 at the Anticoagulation Clinic at the Charlotte Maxeke Johannesburg Academic Hospital (CMJAH) and National Health Laboratory Service in Johannesburg, South Africa. Written informed consent was obtained from all study participants, and the study protocol was approved by the Institutional Review Board (M-230809).

The study included consecutive women, aged 18–45 years old, who were prescribed one of the following oral anticoagulants: Rivaroxaban 20 mg, Rivaroxaban 10 mg, Warfarin (international normalised ratio, INR 2–3) or Warfarin (INR 2.5–3.5). A bleeding, menstrual, gynaecological and obstetric history was obtained and the following were excluded: pregnant women, postmenopausal women with premature ovarian failure, women with laboratory confirmed bleeding disorders, women with cervical or uterine preneoplastic lesions or active malignancy and women with creatinine clearance of <30 mL/min or liver disease.

### Data Collection

At enrolment the following data was collected on predesigned data collection sheets: demographic characteristics, indications for anticoagulation, current anticoagulant therapy, dose and duration, and concomitant medications. A medical history of minor, clinically relevant non major and major bleeding while on anticoagulation was collected. A menstrual history over one-cycle was collected, namely the type of sanitary products and the average length of the menstrual cycle prior to commencing anticoagulation.

Participants completed a MBQ using the MBQ tool developed and validated by Matteson for one menstrual cycles.^19^ The MBQ consists of 20 questions regarding bleeding symptoms, menstrual pain, and quality of life. The responses were added to obtain a minimum score of 0 and a maximum score of 75. Participants self-completed a PBAC daily over one menstrual cycle during the study using the PBAC tool developed by Higham et al and subsequently modified by Spence et al^20,21^ Participants were required to record the number of sanitary products used daily and the degree to which these were soaked on a PBAC, which provided a visual reference. This was returned at completion. The total menstrual blood loss in millilitres was estimated by dividing the total PBAC score by 1.25. According to this visual score, a PBAC score of 100 equates to 80 ml of menstrual blood loss indicating heavy menstrual bleeding. Management of heavy menstrual bleeding as well as supporting laboratory investigations were documented from record review. Iron deficiency was defined according to diagnostic criteria: ferritin of <30 μg/L or transferrin saturation of <20%, and ferritin of 30–100 μg/L.^22^

### Data Analysis

Considering an estimated incidence of heavy menstrual bleeding of 65% in women on anticoagulation, a sample size of 60 was calculated at a CI of 90% using Stata software (Texas, USA).^1,6^ Data was analysed using Statistica 13.2 software (California, USA). Normally distributed, continuous data was presented as mean ± SD and variables with non-Gaussian distribution as median [interquartile range (IQR)]. Comparisons for numerical measurements between subgroups were performed using a parametric unpaired t-test or non-parametric Mann-Whitney test. Comparisons for categorical measurements were performed using chi-square test or Fisher's exact test when necessary. Univariate logistic regression analysis was conducted to identify risk factors for heavy menstrual bleeding with the correspondent unadjusted OR and 95% CI. Significance level was set at <0.05.

## Results

### Clinical and Laboratory Characteristics

**Figure 1.**
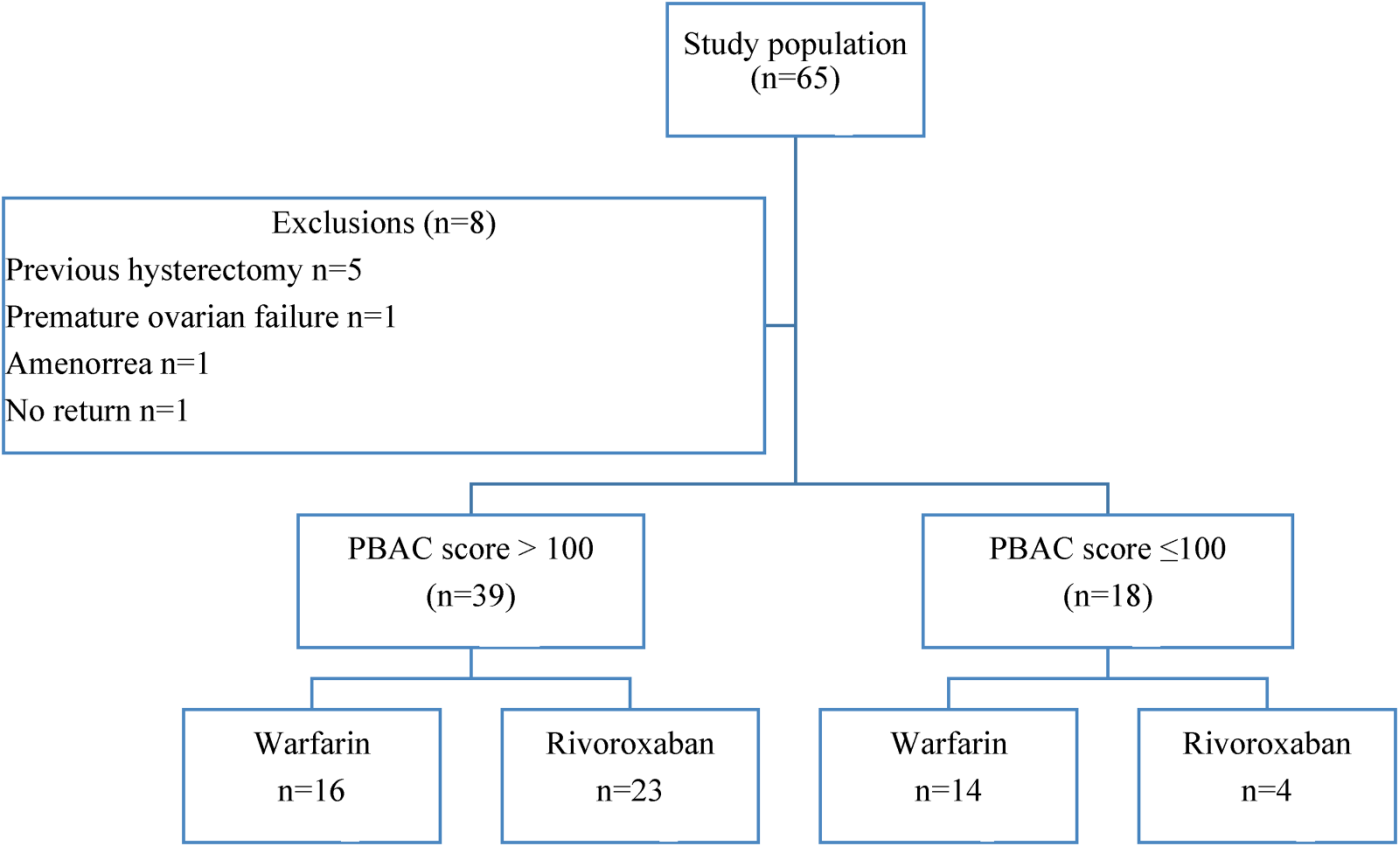
Flow diagram of study participants.

During the study 65 women on anticoagulation were interviewed of whom 57 were eligible for inclusion (Figure 1). The baseline demographics and clinical characteristics of the study participants are described in Table 1. The majority of the women (median [IQR] age of 39 [8] years) were of African ethnicity (n = 52, 91.2%) in keeping with the demographics of the population served by CMJAH. VTE (n = 31, 54.4%) was the most common indication for anticoagulation, followed by valvular heart disease (n = 24, 42.1%). At enrolment, the median duration of anticoagulation was 15.5 [78.0] months. Fifteen (26.3%) women were living with HIV, consistent with the national rates of infection. There were 35 (61.4%) participants on concomitant medications. No participants were receiving concomitant antiplatelet therapy. There were 27 (90.0%) participants diagnosed with iron deficiency on iron studies, performed in 30 participants (Table 2).

**Table 1.** Baseline Characteristics of Study Population.

Demographics	n = 57
Age at study entry (years), median [IQR]	39.00 [8.00]
Ethnicity	
African (n,%)	52 (91.22%)
Non- African (n,%)	5 (8.77%)
**Characteristics**	
Anticoagulant	
Warfarin (n,%)	30 (52.63%)
Rivaroxaban (n,%)	27 (47.37%)
Indication for anticoagulation	
Venous thrombo-embolic disease (n,%)	31 (54.39%)
Valvular heart disease (n,%)	24 (42.10%)
Non- valvular heart disease (n,%)	2 (3.51%)
Duration of anticoagulation (months), median [IQR]	15.50 [78.00]
Co-morbidities	
Cardiac conditions (n,%)	6 (10.5%)
Autoimmune conditions (n,%)	4 (7.02%)
Neurological conditions (n,%)	2 (3.50%)
HIV infected (n,%)	15 (26.32%)
Concomitant medications (n,%)^1^	35 (61.40%)

Key: IQR, Inter- quartile range, HIV, Human Immunodeficiency virus.

^1^Of the participants on warfarin, no participants were receiving CYP2C9 inhibitors.

Of the participants on Rivaroxaban, no participants were receiving CYP3A4 inducers.

**Table 2.** Laboratory Characteristics on Anticoagulation.

Laboratory Characteristics	
Haemoglobin (g/L), mean ± SD (ref: 116–164)	111.18 ± 21.57
Mean cell volume (fL), mean ± SD (ref: 78.9–98.5)	85.91 ± 8.66
Mean corpuscular haemoglobin concentration (g/dL), median [IQR] (ref: 32.7–34.9)	31.60 [2.50]
Serum iron^1^ (µmol/L), mean ± SD (ref: 9–30.4)	8.67 ± 5.46
Transferrin^1^ (g/L), median [IQR] (ref: 2.5–3.8)	3.80 [8.10]
% saturation^1^, median [IQR] (ref: 15–50)	3.75 [9.10]
Ferritin^1^ (µg/l), median [IQR] (ref: 15–150)	34.50 [28.00]
Estimated glomerular filtration rate (mL/min), mean ± SD (ref: 71–121)	59.88 ± 0.71
International normalised ratio on warfarin, mean ± SD	
Range 2.0–3.0	2.51 ± 0.83
Range 2.5–3.5	2.43 ± 0.78

Key: IQR, Inter- quartile range, SD, standard deviation.

1 in 30 participants.

### Menstrual Cycle Characteristics and Impact on Quality of Life

The median cycle length was 6 [4] days on anticoagulation as compared to 5 [2] days prior to commencing anticoagulation (p < 0.001). The median total estimated menstrual blood loss was 120.0 [160.0] ml and the median PBAC score was 155.0 [200.0] in the study population. In participants with iron deficiency (n = 27), the median PBAC score was 171.0 [221.0]. Thirty-nine (68.4%, 95% CI 0.55–0.79) women experienced heavy menstrual bleeding, defined as a PBAC score of >100. Median cycle length on anticoagulation and the MBQ score were significantly higher among women with a PBAC score of >100 (Table 3). The PBAC score correlated positively with the MBQ score (r = 0.547, p < 0.001). No significant difference in median PBAC scores was observed with anticoagulation categorised according to duration of treatment of ≤ 6 months and >6 months (p = 0.198).

**Table 3.** Characteristics of Participants with Pictorial Blood Loss Assessment Chart Scores > 100 and ≤ 100.

Variable	PBAC > 100 n = 39	PBAC ≤100 n = 18	p value
Age (years), median [IQR]	39.00 [7.00]	40.50 [10.00]	0.916
Menstrual cycle length before anticoagulation (days), median [IQR]	5.00 [3.00]	4.00 [1.00]	0.153
Menstrual cycle length on anticoagulation (days), median [IQR]	7.00 [5.00]	5.00 [2.00]	0.002
Duration on anticoagulation (months), median [IQR]	15.00 [70.00]	60.00 [126.00]	0.196
MBQ Score, median [IQR]			
	26.00 [22.00]	9.50 [13.00]	<0.001
Haemoglobin (g/L), mean ± SD	111.23 ± 23.21	110.23 ± 18.23	0.874
% saturation, median [IQR]	3.75 [16.40]	3.72 [3.10]	0.895
Ferritin (µg/l), median [IQR]	34.00 [29.00]	40.00 [35.00]	0.333

Key: IQR, Inter- quartile range, SD, standard deviation, MBQ, menstrual bleeding questionnaire, PBAC, pictorial blood loss assessment chart.

Median total estimated menstrual blood loss, PBAC scores and MBQ scores were higher among participants on Rivaroxaban as compared to Warfarin (p < 0.001) (Table 4). Median cycle length on Rivaroxaban was 9.00 [6.00] days as compared to 5.00 [1.00] days on Warfarin (p < 0.001). Twenty-three (85.2%) of the participants on Rivaroxaban and 16 (53.3%) of the participants on Warfarin had PBAC scores of >100 (p < 0.010). There was no difference in haemoglobin levels and iron studies according to anticoagulant groups. Iron deficiency was diagnosed in 12 (40.0%) participants on Rivaroxaban for a median of 8.5 [12.0] months and 15 (50.0%) participants on Warfarin for a median of 84.0 [86] months.

**Table 4.** Characteristics of Participants on Warfarin and Rivaroxaban.

Variable	Warfarin n = 30	Rivaroxaban n = 27	p value
Age (years), median [IQR]	38.50 [10.00]	39.00 [7.00]	0.471
Menstrual cycle length before anticoagulation (days), median [IQR]	5.00 [1.00]	5.00 [2.50]	0.418
Menstrual cycle length on anticoagulation (days), median [IQR]	5.00 [1.00]	9.00 [6.00]	<0.001
Duration on anticoagulation (months), median [IQR]	80.00 [114.00]	8.00 [10.50]	<0.001
Haemoglobin (g/L), mean ± SD	108.22 ± 18.60	114.02 ± 24.08	0.323
Serum iron (µmol/L), mean ± SD	8.77 ± 5.71	8.57 ± 5.41	0.922
% saturation (%), median [IQR]	4.00 [9.00]	3.43 [13.50]	0.868
Ferritin (µg/l), median [IQR]	30.00 [37.00]	39.00 [30.00]	0.934
Blood loss (ml), median [IQR]	83.20 [68.80]	193.60 [157.60]	<0.001
PBAC Score, median [IQR]	104.00 [86.00]	242.00 [197.00]	<0.001
MBQ score, median [IQR]	10.00 [13.00]	29.50 [13.50]	<0.001

Key: IQR, Inter- quartile range, SD, standard deviation, MBQ, menstrual bleeding questionnaire, PBAC, pictorial blood loss assessment chart.

On univariate analysis, the odds of heavy menstrual bleeding increased approximately 5-fold (OR 5.03, 95% CI 1.40–18.12, p < 0.014) with Rivaroxaban (Supplementary Table 1).

**Figure 2.**
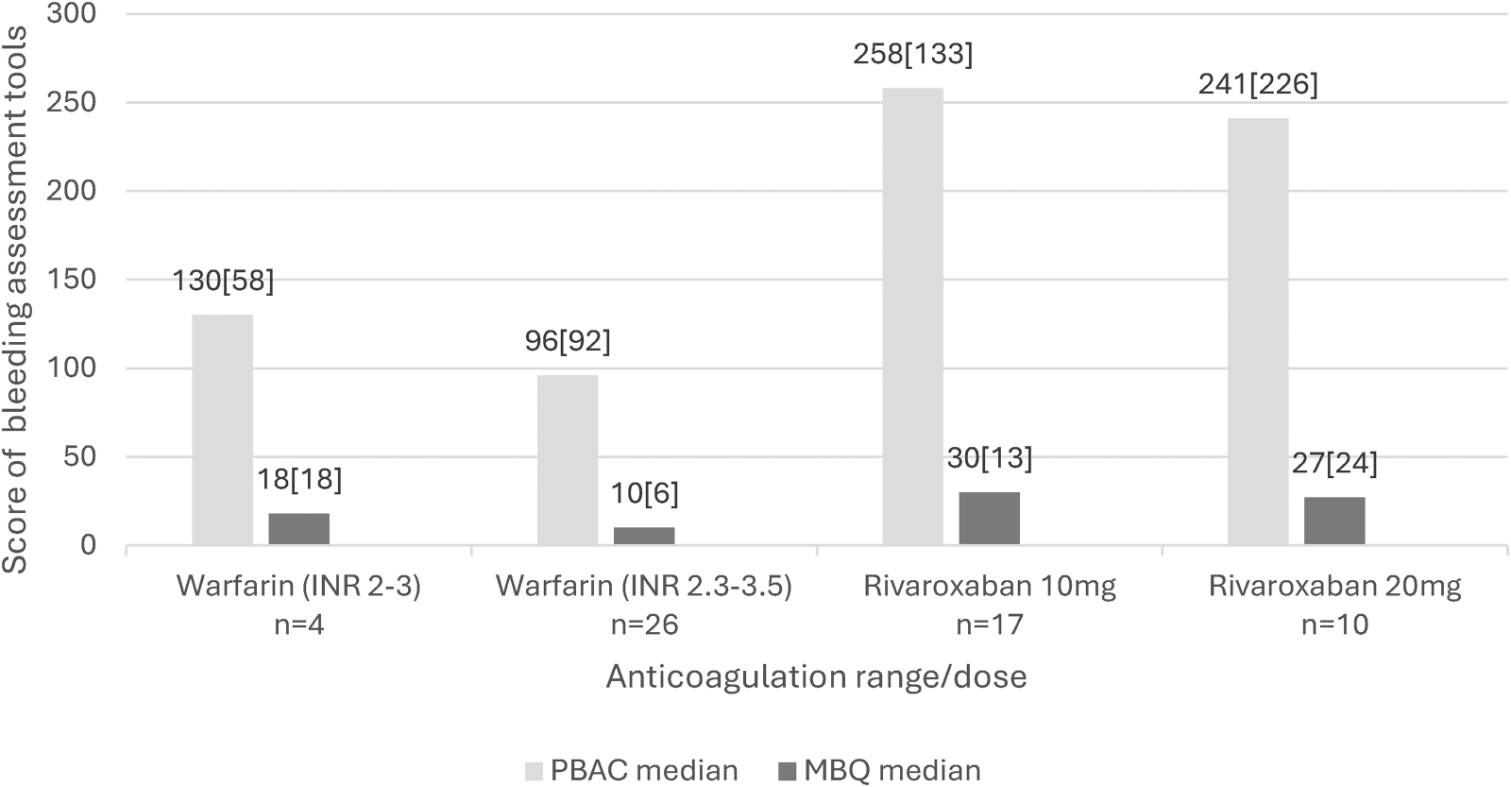
Median PBAC and menstrual bleeding questionnaire scores according to anticoagulant range/dose.

Figure 2 describes the PBAC and MBQ scores according to Warfarin INR range of 2–3 and 2.5–3.5 and Rivaroxaban dose of 10 mg and 20 mg. Median cycle length on Warfarin INR 2–3 and 2.5–3.5 was 6.00 [3.00] and 5.00 [2.00] days respectively. Median cycle length on Rivaroxaban 10 mg and 20 mg was 9.00 [4.00] and 10.00 [10.00] days respectively.

Median MBQ scores of impact on quality of life were significantly higher among participants on Rivaroxaban as compared to Warfarin for all 20 questions except question 19 on predictability of menstrual period start date (Supplementary Table 2). The key factors impacting on quality of life are described in Table 5. Employed women on Rivaroxaban (12, 50.0%) were more likely to miss days of work because of menstrual bleeding as compared to Warfarin (0, 0.0%) (p < 0.001). There were 2 (8.33%) women on Rivaroxaban who reported missing >13 days of work.

**Table 5.** Menstrual Bleeding Questionnaire Factors Impacting on Women's Quality of Life, According to Warfarin and Rivaroxaban.

Factor	Total n = 57	Warfarin n = 30	Rivaroxaban n = 27	p value
Changing sanitary products at night, during sleep (MBQ Q5 score ≥1) (n, %)	23 (40.35%)	7 (23.33%)	16 (59.26%)	0.006
Passing of blood clots staining clothing (MBQ Q7 score ≥1) (n, %)	37 (64.91%)	13 (43.33%)	24 (88.89%)	<0.001
Pain related to menstrual period (MBQ Q8 score ≥1) (n, %)	27 (47.37%)	8 (26.67%)	19 (70.37%)	0.001
Difficulty with work because of menstrual bleeding (MBQ Q10 score ≥1) (n, %)	21 (36.84%)	5 (16.67%)	16 (59.26%)	0.002
No. of days avoiding family activities (MBQ Q12 score ≥1) (n, %)	22 (38.60%)	5 (16.67%)	17 (62.96%)	0.003
No. of days avoiding social activities (MBQ Q14 score ≥1) (n, %)	16 (28.07%)	5 (16.67%)	11 (40.74%)	0.141
Extreme concern about blood staining clothes (MBQ Q18, scale 0–10), median [IQR]	2.00 [6.80]	2.00 [2.00]	7.00 [7.00]	<0.001
Not being able to predict at all when period will end (MBQ Q20 score >1)	21 (36.84%)	5 (16.67%)	16 (59.26%)	0.001

Key: IQR, Inter- quartile range, MBQ, menstrual bleeding questionnaire.

### Management

On record review, contraception included barrier methods (n = 26, 45.6%), intra-uterine contraceptives, (n = 5, 8.8%), subdermal implants (n = 5, 8.8%), contraceptive injection, (n = 5, 8.8%), and combined oral contraceptive (n = 5, 8.8%).

Twenty-nine (74.4%) participants on anticoagulation received management for heavy menstrual bleeding including its complication of iron deficiency (Table 6). The majority of participants with heavy menstrual bleeding were managed as out-patients and 3 (10.3%) required hospitalisation for red cell transfusions and/or tranexamic acid. In 8 (27.6%) women the anticoagulant was changed from Rivaroxaban to Warfarin (n = 5) or Apixaban (n = 3); following which 6 (75.0%) reported an improvement of symptoms. In a further 4 (13.8%) the dose of Rivaroxaban was reduced and anti-Xa levels monitored. This resulted in an improvement of symptoms in half. In 4 (13.8%) women referred to gynaecology for the management of heavy menstrual bleeding, symptoms were controlled with hormonal contraception alone.

**Table 6.** Management of Heavy Menstrual Bleeding.

Treatment of HMB	Number n = 29
Oral iron supplements (n, %)	23 (79.31%)
Intravenous iron infusion (n, %)	3 (10.34%)
Red cell transfusion (n, %)	3 (10.34%)
Tranexamic acid (n,%)	2 (6.90%)
Change of anticoagulant (n, %)	8 (27.58%)
Dose reduction of anticoagulation (n, %)	4 (13.79%)
Referral to gynaecology (n, %)	4 (13.79%)
Hospital admission (n, %)	3 (10.34%)

Key: HMB, heavy menstrual bleeding.

## Discussion

This single-centre study provides important insights into the impact of heavy menstrual bleeding on quality of life, as determined by validated PBAC and MBQ tools. The findings of this study confirm that in South African women in the reproductive age group on anticoagulant therapy, heavy menstrual bleeding is an important side effect. Approximately two-thirds of women who received anticoagulant treatment for a range of conditions including VTE, valvular and non-valvular heart disease experienced heavy menstrual bleeding. These rates are consistent with earlier studies in different ethnic groups. In the international TEAM-VTE study performed across eight countries in Europe, rates of heavy menstrual bleeding of 66.0%, 95% CI 0.6–0.8 were reported among 98 anticoagulated women.^11^

In the current study the median PBAC score was 155.0 [200.0] in women on anticoagulation for a median duration of 15.5 [78.0] months. This finding is comparable with an earlier study of women on long-term warfarin which reported a median PBAC score of 210.0 (range 14.0-1010.0).^17^ In contrast, the more recent, TEAM-VTE study reported a lower median PBAC score following initiation of anticoagulation of 95.0 [221.0] for the first cycle.^11^ Moreover, the authors observed a non-significant decline in the rates of heavy menstrual bleeding over the follow-up six-month period. Future studies are indicated over consecutive menstrual cycles to assess the impact of anticoagulation beyond six-months on heavy menstrual bleeding and its impact on quality of life.

Importantly, heavy menstrual bleeding was associated with a considerable negative impact on quality of life including changing sanitary products at night, passing of blood clots staining clothing, pain, difficulty with work, avoiding family and social activities, extreme concern about blood staining clothes and not being able to predict when the menstrual cycle will end. Mental and social well-being are important aspects of total health care. It is thus essential for clinicians to take a menstrual history and provide adequate counselling when prescribing and monitoring anticoagulant therapy. The high PBAC and MBQ completion rates in this study suggest that these are useful validated tools which take into consideration cultural differences.

Consistent with earlier observations, the rates of heavy menstrual bleeding and its negative impact on quality of life were most pronounced among women receiving Rivaroxaban.^8,11^ While the mechanism has not been described, it is possible that factor Xa inhibitors induce bleeding by compromising uterine mucosal integrity similar to the effect on the gastrointestinal mucosa. Rivaroxaban was associated with a 5-fold increased risk of heavy menstrual bleeding, with prolongation of the menstrual cycle and an increased need for modification of anticoagulant treatment as compared to Warfarin. This study consisted of a large subgroup of women on Warfarin (52.6%) in contrast to studies from high income countries. This study describes the heavy menstrual bleeding characteristics of 24 (42.1%) women with valvular heart disease who required an INR of 2.5–3.5, which were similar to earlier studies on Warfarin.^12,17^ The increasing use of DOACs in our setting, owing to the lower risk profile and ease of use, requires careful consideration in women in the reproductive age group. Moreover, owing to the reduced monitoring requirements of Rivaroxaban, symptoms and complications of heavy menstrual bleeding may be identified later as compared to Warfarin. In 12 (30.8%) women with heavy menstrual bleeding Rivaroxaban treatment was modified by reducing the dose or switching to Warfarin or Apixaban. The results of the EINSTEIN-CHOICE sub-study, suggest that in women with heavy menstrual bleeding, the dose of Rivaroxaban can be safely reduced to 10 mg daily for extended treatment of VTE after completion of 6 to 12 months of anticoagulation.^23^ Rivaroxaban 10 mg was associated with a decrease in cycle length and heavy menstrual bleeding duration as compared to 20 mg. With the availability of several anticoagulant agents, switching agents is another reliable option.^6^ The ongoing heavy MEnstrual bleeding in premenopausal women treated with DirEct oral Anticoagulants (MEDEA) trial is a Dutch multicentre, randomized trial in women with heavy menstrual bleeding on factor Xa inhibitor treatment. The study is currently randomising women to either switch to dabigatran, continue on the factor Xa inhibitor with the addition of an antifibrinolytic agent or to continue on the factor Xa inhibitor with no intervention.^24^ In particular, the anti-fibrinolytic agent tranexamic acid has not been shown to increase the risk of thrombosis and has been associated with a decrease in heavy menstrual bleeding.^25^ Recent phase 2 trials of Factor XI inhibitors have reported a reduction in major or clinically relevant non-major bleeding compared to prophylactic LMWH in orthopaedic patients and DOAC in non-valvular atrial fibrillation. These promising early results have led to the initiation of phase 3 trials with safety results awaited.^26,27^

Women who present with anticoagulant associated heavy menstrual bleeding require an individualised management approach in consultation with gynaecology. Treatment options in pre-menopausal women also include prescribing hormone contraception and uterine surgical interventions.^16,28^ Medical management for heavy menstrual bleeding was initiated in 74.4% (n = 29) of which 13.8% (n = 4) were referred to gynaecology for hormonal contraception. Levonorgestrel intrauterine systems are safe and effective and have been associated with blood loss reduction by 80%, improved compliance and low side effect profiles and as such are the preferred modality.^29^ They result in amenorrhea in 50% of women. The etonogestrel subdermal implant results in amenorrhea in approximately 20% of women and depot-medroxyprogesterone acetate results in amenorrhea in greater than 50% of women. Furthermore, the combined oral contraceptive is an alternative which has not been associated with a significantly increased risk of recurrent thrombosis in women on anticoagulation therapy.^13^

Our results must however be interpreted in light of certain limitations. First, the PBAC and MBQ were assessed over one menstrual cycle. Nonetheless, the PERIOD study which assessed PBAC and MBQ scores over two cycles did not show a significant difference between cycles one and two.^8^ Second, subgroup analysis according to the range of INR and dose of Rivaroxaban could not be assessed owing to the small number of participants in particular with an INR range of 2.0–3.0. Third, iron studies were available in only half of the study participants which can be attributed to the underreporting of symptoms of heavy menstrual bleeding. Also, laboratory parameters before the start of anticoagulant therapy were not collected. Fourth, cycle length prior to starting anticoagulation was determined retrospectively. Recall bias, in particular for patients on treatment for > 6 months (n = 42, 73.7%), could have resulted in an inaccurate assessment of prior cycle length. Nonetheless previous studies performed at initiation of anticoagulation, showed similar median cycle lengths.^7,8^ Finally, the study employed the semi-quantitative (PBAC) assessment to quantify menstrual blood loss. The PBAC, however, is widely used in clinical studies and has been associated with a sensitivity of 58–99% and a specificity 75–89% for scores above 100 for heavy menstrual bleeding.^30^

## Conclusion

In conclusion, this study adds to the evidence that heavy menstrual bleeding is a frequent complication during anticoagulation therapy, which was associated with a considerable negative impact on quality of life. This was most significant for women on Rivaroxaban as compared to Warfarin. The majority of participants with heavy menstrual bleeding were managed medically as out-patients. It is thus important for anticoagulation clinics to incorporate into daily practice counselling the possible changes in the menstrual cycle length and bleeding, monitoring and appropriate treatment for heavy menstrual bleeding and iron deficiency. Owing to the observational study design and small number of participants on subgroup analysis according to anticoagulant ranges/doses, the results of ongoing randomized trials are awaited to assess the optimal interventions for heavy menstrual bleeding in this group of patients.

## Supplemental Material

sj-docx-1-cat-10.1177_10760296241281366 - Supplemental material for Incidence and Impact on Quality of Life of Heavy Menstrual Bleeding in Women on Oral Anticoagulant Therapy Supplemental material, sj-docx-1-cat-10.1177_10760296241281366 for Incidence and Impact on Quality of Life of Heavy Menstrual Bleeding in Women on Oral Anticoagulant Therapy by Naseerah Hassan, Elise Schapkaitz and Haroun Rhemtula, Nolukholo Ncete in Clinical and Applied Thrombosis/Hemostasis
