# Supplementary material for: Incidence and Impact on Quality of Life of Heavy Menstrual Bleeding in Women on Oral Anticoagulant Therapy
Source: Clin Appl Thromb Hemost. 2024 Aug 30;30:10760296241281366. doi: 10.1177/10760296241281366 (PMC11367687; doi:10.1177/10760296241281366)
Supplement: sj-docx-1-cat-10.1177_10760296241281366 - Supplemental material for Incidence and Impact on Quality of Life of Heavy Menstrual Bleeding in Women on Oral Anticoagulant Therapy [file sj-docx-1-cat-10.1177_10760296241281366.docx]

Supplementary tables

Table 1S Univariate logistic regression analysis of heavy menstrual bleeding

| **Variable**   \|  \| \| --- \| | **OR** | **95% CI** | **p value** |
| --- | --- | --- | --- | --- |
| Duration on anticoagulation | 0.73 | 0.20 -2.70 | 0.634 |
| \| Previous history of bleeding \| \| --- \| | 2.80 | 0.69-11.37 | 0.150 |
| \| Contraception use \| \| --- \| | 1.00 | 0.30-3.32 | 1.000 |
| Co-morbidities | 1.04 | 0.34-3.19 | 0.060 |
| Rivaroxaban anticoagulation | 5.03 | 1.40-18.12 | 0.014 |
| Warfarin high range (2.5-3.5) | 0.33 | 0.03-3.37 | 0.368 |
| Rivaroxaban standard dose (20mg) | 0.15 | 0.013-1.66 | 0.121 |
| Key: CI, confidence interval, OR, odds ratio | | | |

Table 2S Median menstrual bleeding questionnaire scores according to Warfarin and Rivaroxaban

| **Factor** | **Total**  **n=57** | **Warfarin**  **n=30** | **Rivaroxaban**  **n=27** | **p value** |
| --- | --- | --- | --- | --- |
| Description of menstruation (light to heavy) (MBQ Q1, scores 0-4) median [IQR] | 3.00 [2.00] | 2.00 [2.00] | 3.00 [1.50] | 0.008 |
| No. of sanitary products soaked (MBQ Q2, scores 0-5) median [IQR] | 2.00 [1.00] | 1.00 [2.00] | 2.00 [1.00] | <0.001 |
| Wearing an incontinence brief or more than one sanitary product at a time (MBQ Q3, scores 0-5) median [IQR] | 1.00 [1.00] | 0.00 [1.00] | 1.00 [2.00] | 0.008 |
| Soaking through outer clothes (MBQ Q4, scores 0-3) median [IQR] | 0.00 [1.00] | 0.00 [0.00] | 1.00 [1.00] | <0.001 |
| Changing sanitary products at night, during sleep (MBQ Q5, scores 0-3) median [IQR] | 0.00 [1.00] | 0.00 [0.00] | 1.00 [1.00] | 0.006 |
| Passing of blood clots (MBQ Q6, scores 0-3) median [IQR] | 1.00 [2.00] | 0.00 [1.00] | 2.00 [1.00] | <0.001 |
| Passing of blood clots staining clothing (MBQ Q7, scores 0-3) median [IQR] | 1.00 [1.00] | 0.00 [1.00] | 1.00 [1.00] | <0.001 |
| Pain related to menstrual period (MBQ Q8, scores 0-3) median [IQR] | 0.00 [2.00] | 0.00 [1.00] | 1.00 [2.00] | 0.006 |
| Duration of period in weeks over last month (MBQ Q9, scores 0-3) median [IQR] | 1.00 [1.00] | 0.00 [1.00] | 1.00 [1.50] | 0.035 |
| Difficulty with work because of bleeding (MBQ Q10, scores 0-4) median [IQR] | 0.00 [1.00] | 0.00 [0.00] | 1.00 [2.00] | 0.001 |
| No. of work days missed due to bleeding (MBQ Q11, scores 0-4) median [IQR] | 0.00 [0.00] | 0.00 [0.00] | 0.50 [1.00] | <0.001 |
| No. of days avoiding family activities (MBQ Q12, scores 0-4) median [IQR] | 0.00 [1.00] | 0.00 [0.00] | 1.00 [0.00] | 0.001 |
| Carrying extra sanitary products (MBQ Q13, scores 0-2) median [IQR] | 0.00 [1.00] | 0.00 [0.00] | 2.00 [1.00] | <0.001 |
| No. of days avoiding social activities (MBQ Q14, scores 0-4) median [IQR] | 0.00 [1.00] | 0.00 [0.00] | 0.00 [1.00] | 0.042 |
| Planning activities around availability of a bathroom (MBQ Q15, scores 0-4) median [IQR] | 0.00 [1.00] | 0.00 [0.00] | 1.00 [0.00] | 0.001 |
| Carrying extra clothes in case of staining (MBQ Q16, scores 0-3) median [IQR] | 0.00 [1.00] | 0.00 [0.00] | 1.00 [2.00] | <0.001 |
| Choice of clothing depending on bleeding (MBQ Q17, scores 0-4) median [IQR] | 1.00 [1.00] | 0.00 [1.00] | 1.00 [2.00] | 0.013 |
| Extreme concern about blood staining clothes (MBQ Q18, scale 0-10), median [IQR] | 2.00 [7.00] | 2.00 [2.00] | 7.00 [7.00] | <0.001 |
| Not being able to predict when period will start (MBQ Q19, scores 0-2) median [IQR] | 1.00 [1.00] | 1.00 [0.00] | 1.00 [1.00] | 0.072 |
| Not being able to predict at all when period will end (MBQ Q20, scores 0-2) median [IQR] | 1.00 [1.00] | 1.00 [0.00] | 2.00 [1.00] | <0.001 |
| Key: IQR, Inter- quartile range, MBQ, menstrual bleeding questionnaire | | | | |
